# Activating Cancer Hallmarks through Changes in mRNA/Protein Regulation

**DOI:** 10.1021/acs.jproteome.4c00284

**Published:** 2025-07-17

**Authors:** Jose Humberto Giraldez Chavez, Nathaniel Barton, Caleb M Lindgren, Bryn Mendenhall, Benjamin Kimball, Samuel H Payne

**Affiliations:** Biology Department, 6756 Brigham Young University, Provo, Utah 84602, United States

**Keywords:** bioinformatics, multiomics, cancer, proteogenomics, data analysis

## Abstract

As a diverse family of diseases, cancer is unified by a set of common dysfunctions, such as limitless growth potential and an insensitivity to antigrowth signals. These shared overarching biological processes have been termed the hallmarks of cancer. To better understand the root cause of cellular dysregulation, intense molecular characterization of tumors has utilized DNA, RNA, and protein measurement techniques to produce proteogenomic data. In large cancer cohort studies, genomic and proteogenomic data have frequently identified many cancer hallmarks including cell cycle and cell signaling. However, altered metabolism, a known cancer hallmark, is not as clearly identified in mutation screens or differential expression analyses. Here, we introduce a new computational method to identify changes in cellular regulation by focusing on the mRNA/protein relationship. We create a metric, Δ_corr, to capture when the mRNA/protein correlation changes significantly between tumor and normal tissues and show that it is distinct from differential expression and also not associated with DNA mutation profiles. Our method clearly highlights altered metabolic pathways across multiple tumor types. Δ_corr gives researchers a new perspective on the dysfunction of tumor cells and introduces a novel method for proteogenomic data integration.

## Graphical abstract

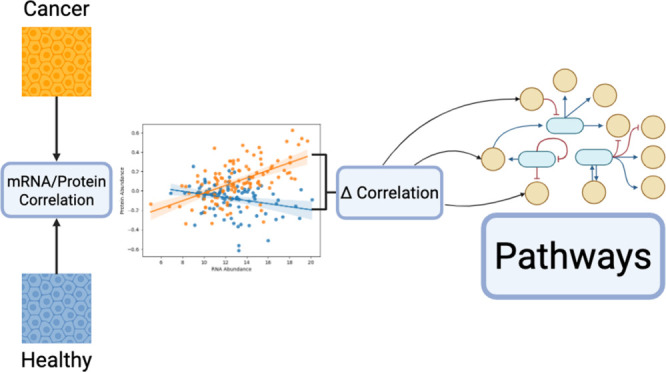

## Introduction

Cancer is a disease of cellular dysfunction. As described by Hanahan and Weinberg,^1,2^ these dysfunctions typically fit into one of a few broad categories called hallmarks. After the discovery that cancer was caused by DNA mutation,^3^ early molecular analysis of tumors focused on DNA sequencing to identify common driver mutations. Extensive genomic characterization of tumors has identified DNA driver mutations, which help identify disease subtypes and guide precision therapy.^4,5^ Although useful, this catalog of DNA mutations does not describe the altered cellular state that defines the tumor phenotype. For this reason, many cancer studies create proteogenomic data^6,7^ to better link somatic mutations to changes in mRNA and/or protein abundance.

Although mRNA is only a temporary intermediary between genome and protein, it is frequently measured and studied as a proxy for the cellular state due to its convenient and robust data acquisition methods. However, a large body of evidence shows that mRNA abundance has a low correlation to protein abundance,^8–11^ due to translational and post-translational regulation.^12–17^ For proteogenomic data that contains both mRNA and protein abundance, a common data integration exercise is to predict protein abundance from mRNA,^18^ often with only modest accuracy. In cancer studies,^19–22^ although many genes have a correlation >0.6, a large number of genes have correlations <0.2, including negative correlation values. Pathway analyses consistently discovered that these patterns of mRNA/protein correlation – including high and low correlations – are biologically coherent; metabolic and signaling pathways are enriched in genes with a high correlation while ribosomes and oxidative phosphorylation are enriched in genes with a low/negative correlation.

Integrative data analysis of mRNA and proteins can also be used to investigate biological hypotheses. For example, does the relationship of mRNA and protein change in a disease? Is there an altered protein homeostasis in cancer? As cancer is marked by regulatory dysfunction, it is intriguing to evaluate whether the regulatory relationship between mRNA and proteins has changed in tumor cells. Indeed, a fundamental and unexplored question is whether mRNA/protein quantitative relationships are static or dynamic. A static relationship implies that the mRNA/protein correlation is the same in all tissues and disease states. This is an implicit assumption of algorithms that learn to predict protein abundance from mRNA, as they are trained on data from a limited set of conditions. Although some have suggested that this relationship is fixed and static,^23,24^ several studies demonstrate that this can be a fluid relationship.^25–27^ For example, Takemon et al. discovered age-dependent changes in protein concentration in the absence of corresponding changes in mRNA.^28^ Indeed, a change in any post-translational regulatory mechanism would create a dynamic mRNA/protein relationship. The extent of such changes in cancer is unknown.

The stability of the mRNA/protein relationship remains underexplored, largely due to the absence of a sufficiently powered data set. To characterize the correlation, a data set must have sufficient samples in multiple tissues and measure both protein and mRNA for thousands of genes. The Clinical Proteomic Tumor Analysis Consortium (CPTAC) has created one such data set, with proteogenomic data for ∼100 tumor samples per tumor type.^29,30^ It also contain matched normal tissues for many tumor samples. Given the complex regulatory mechanisms utilized by cells and the growing evidence of regulatory plasticity in cancer cells, we sought to understand the mRNA/protein relationship in cancer tissues. Specifically, we examined whether this relationship is static or fluid and which cancer related genes and pathways are affected. We discover major regulatory changes across thousands of genes that lead to the activation of pathways associated with hallmarks of cancer.

## Methods

### Publicly Accessible Analysis

All of the data analysis scripts used to generate figures and metrics in this publication are publicly accessible in our GitHub repository at https://github.com/PayneLab/pancancerProteinMRNA. Individual scripts are listed in various sections below.

### Data Sets

We utilized the CPTAC data from the pan-cancer resource API.^30^ Specifically, we used the data for transcriptomics, proteomics and DNA somatic mutation, accessed through the publicly available CPTAC Python API.^29^ Data generation and data analysis methods for these are detailed in the linked publications. Briefly, the transcriptomics data is RNASeq gathered on all samples (tumor and normal), and TMT-labeled global proteomics data was collected to characterize the proteome (tumor and normal). A direct link to the harmonized data tables stored at the Proteome Data Commons is https://pdc.cancer.gov/pdc/cptac-pancancer. According to the published studies, all of the studies were performed with proper ethics.

### Gene-wise Correlations

To identify the relationship between mRNA and proteins in the cancer cohorts, we calculated the Spearman correlations between transcriptomic and proteomic data by patient, separately, for tumor and normal samples. A minimum sample size cutoff of 15 data points per gene was enforced, with the exception of endometrial cancer, which has a cutoff of 10 due to having a smaller number of normal samples. The exact implementation of this correlation can be found in our GitHub repository at Implementation is here (∼/notebook_steps/data/Make_Tumor-Normal_Correlation_Dataframe.ipynb). Data corresponding to the mRNA/protein correlation of each healthy and tumor genes for each of the cancer cohorts are here (∼/notebook_steps/data/tumor_normal_correlation_df.csv).

Additionally, using CPTAC’s clinical data, we can analyze the cancer stage for each sample. We combined the stage clinical information and joined it with each patient’s proteomic and transcriptomic data. We then grouped samples by cancer, gene, and stage in a single data frame. Then, we did a Spearman correlation between normal and tumor gene transcriptomics and proteomics of the genes that are present in each stage. We set a cutoff for stages that did not have 10 or more genes per stage to keep better consistency. We organized the correlations in each stage and plotted the data into a Kernel density estimation plot (Figure 1). For implementation see:

**Figure 1:**
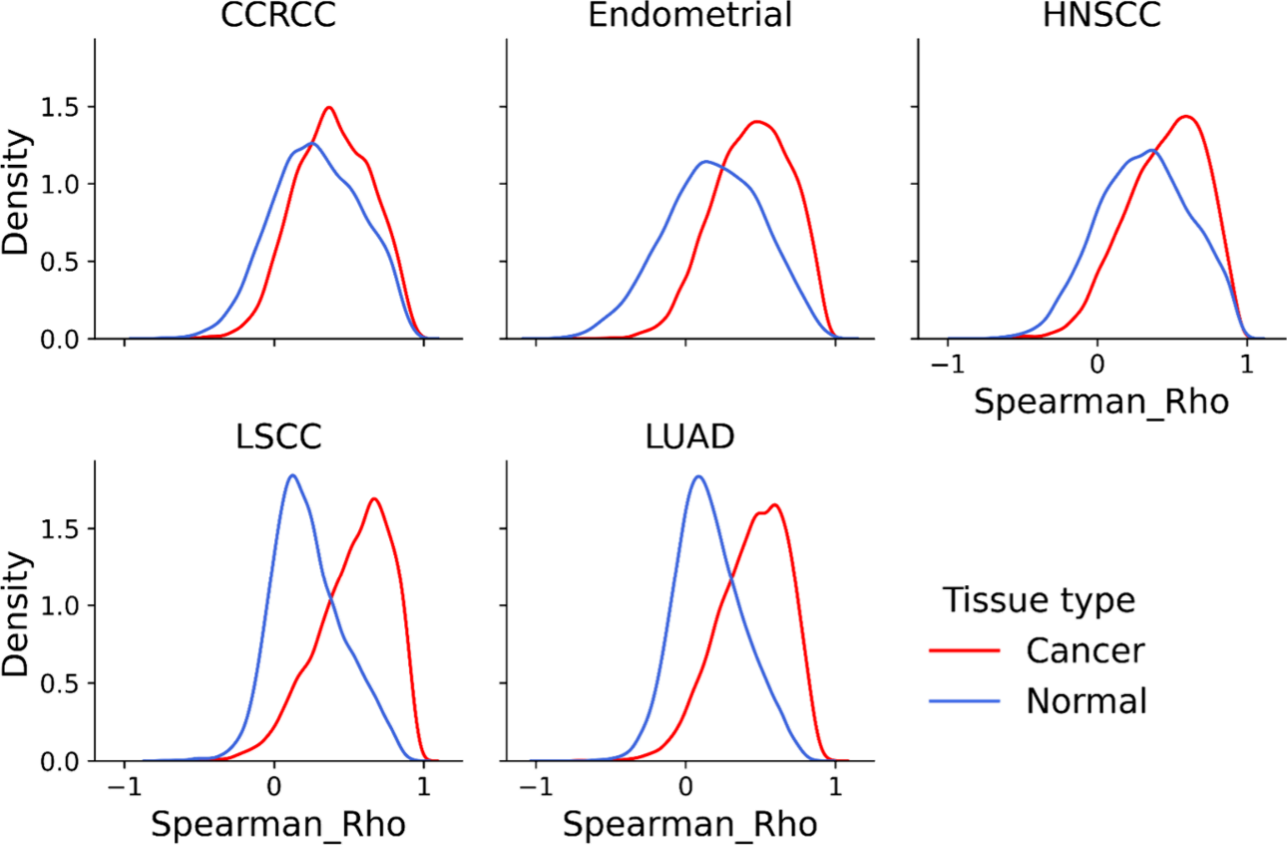
Distribution of mRNA/protein correlations. For each of the five CPTAC cohorts, the Spearman correlation between mRNA and protein was performed for all genes. The distribution of these correlation coefficients is plotted for normal samples (blue) and tumor samples.

∼/notebook_steps/data/Make_Cancer_stages_correlations_df.ipynb and ∼ /notebook_steps/Make_Figure_1_Correlation_Change_by_Cancer_Stage.ipynb

### Correlation Changes

To quantify the change in regulation for a given gene between tumor and normal tissue, we calculated the difference in spearman correlation coefficients, a term we named Δ_corr. For each gene with a minimum of *n* = 15 (*n* = 10 for endometrial) tumor and normal samples with proteomic and transcriptomic data, we first calculated the tumor and normal correlation coefficients (ρ). We then calculated the Δ_corr value using the following equation:

$$\Delta \text{\_}\mathrm{corr}=\rho_{\mathrm{tumor}}-\rho_{\mathrm{normal}}$$

A positive Δ_corr translates to a higher correlation coefficient in tumor samples, while a negative Δ_corr translates to a lower correlation coefficient in tumor samples. For implementation, see the readme found in the GItHub repository at ∼ /notebook_steps.

### Permutation Test

In order to calculate probability values for Δ_corr, we used label permutation. The advantage of label permutation is that it allows us to explicitly create a distribution of values under our null hypothesis and then compare the test statistic to this null distribution. Our null hypothesis is that the mRNA/protein correlation does not change between normal and tumor tissue. This is equivalent to saying that the label of a sample as a tumor or normal is not meaningful when creating an mRNA/protein correlation. Thus, a direct instance of this null hypothesis is one where the data series has a randomized label. By performing many randomized label experiments (i.e., label permutations), we can create a null distribution that exactly matches our null hypothesis.

Using tissue for both normal and tumor samples and its matching gene readings for individual patients, we did a label permutation to create a false correlation. Since all patients have proteomic and transcriptomic gene information divided into normal and tumor samples, we join them in a single data frame to be accessed from. Then, we randomly assigned the labels of normal and tumor to each measurement, keeping the same amount of tumor and normal data points. To provide a more precise procedure, we set a cutoff for genes that have 15 or more valid data points (except endometrial, which has a cutoff of 10 or more). From each data frame that has been permuted, we do a Spearman correlation from the permuted data frame. This gives us a false Δ_corr for permuted data frames as seen in Supplemental Figure 3B. We then repeat 10,000 permutations, each resulting in a false Δ_corr. In Supplemental Figure 3C, we can see how different the original Δ_corr from the permuted delta correlations is. From the list of Δ_corr we obtain a *z*-score with respect to the original nonpermuted Δ_corr. This *z*-score is transformed into a *p*-value showing the significance of the change in terms of a gene's mRNA/protein correlation in a tissue. The p-values were corrected through a Benjamini-Hochberg Procedure to account for the False Discovery Rate (FDR). We do this for all genes and all 5 selected tissues on the BYU supercomputer.

For implementation see:

∼/notebook_steps/data/Scripts_to_Make_Cancer_Delta_Corr_and_P_Value_Dataframe

### Differential Expression

To determine differential expression of a gene’s mRNA and protein abundance between tumor and normal tissue, we performed a Wilcoxon rank-sums test and calculated the mean log2 fold-change for each gene in each cancer type. *P*-values from the Wilcoxon rank-sums test were corrected for multiple hypothesis testing via Benjamini–Hochberg correction. Genes were considered to be differentially expressed if they had a BH p-value <0.05 and |log2 fold-change| > 1, following cutoffs used in.^19^ For implementation see ∼ /notebook_steps/data/Make_Proteomics_and_Transcriptomics_Differential_Expression_Dataframes.ipynb

### Mutation Effects in Trans Genes

To determine the effect of mutations on Δ_corr, we first identified the most frequently mutated genes in each tumor cohort. For each cohort, we identified the top 10 most frequently mutated genes with at least *n* = 15 (*n* = 10 for endometrial) tumor mutant and tumor wild-type samples for statistical significance. For tumor cohorts that did not contain at least 10 mutated genes that met the cutoff, all genes meeting the cutoff were used. For implementation, see ∼ /notebook_steps/data/Scripts_to_make_transmutation_effects_dataframes/Find_Most_Mutated_Genes_and_Write_transmutation_scripts.ipynb

For each of the most frequently mutated genes, we next calculated the Δ_corr for each transgene between the tumor-mutated and tumor-wildtype samples. To determine the significance of the Δ_corr, we performed a label based permutation (see Permutation Test) with 10,000 permutations. The permutation p-values for each mutated gene group were then corrected for multiple hypothesis testing via Benjamini–Hochberg correction. A mutation was considered to have a significant effect on a transgene if the Δ_corr permutation BH *p*-value <0.05. For implementation see ∼ /notebook_steps/data/Scripts_to_make_transmutation_effects_dataframes/transmutation_effects.py

Permutation tests were performed on a BYU supercomputer. For bash scripts ran on the supercomputer, see the readme found in ∼ /notebook_steps

### Pathway Analysis

We used the gProfiler python API to perform a gene set enrichment analysis of significant Δ_corr genes. For the enrichment analysis, we used the genes that have a FDR corrected *p*-value of less than 0.05 and absolute threshold of 0.2. The genes used for background are the set of genes found in the corresponding cancer. We evaluated our genes with the Kyoto Encyclopedia of Genes and Genomes (KEGG) library of pathways. This was repeated for each of our 5 tissues. For implementation, see ∼ /notebook_steps/Make_Figure_5_Cancer_Hallmarks_Pathways.ipynb

## Results

The Clinical Proteomic Tumor Analysis Consortium (CPTAC) creates proteogenomic data for cancer discovery. A typical CPTAC cohort contains 100 tumor samples, which are characterized with genomics, transcriptomics, and proteomics, as well as clinical demographic and treatment information. Five CPTAC cohorts have also characterized normal tissue: lung adenocarcinoma (LUAD), lung squamous cell carcinoma (LSCC), clear cell renal cell carcinoma (ccRCC), endometrial cancer (ENDO), and head and neck squamous cell carcinoma (HNSCC). Therefore, we examined these five tumor types to identify changes in the mRNA-protein relationship between healthy and cancer tissue.

### The mRNA/Protein Regulatory Relationship Is Fluid and Changes between Health and Disease

We performed a gene-wise correlation comparison between the abundance of mRNA and protein for each gene across all samples in a cohort. Each gene’s mRNA/protein relationship is unique^31^ including positive, negative, and noncorrelations (Figure S1). It is often assumed that the lack of correlation is an artifact of poor measurement;^32^ however, the wide range of correlation values has been observed in proteogenomic analysis for the past 20 years through multiple iterations of both mRNA and protein measurement technologies.^20,33,34^ Moreover, as shown in Figure 1, the correlation value is dynamic and changes across tissues and disease states. The large shift in the distribution of correlation values between tumor and normal tissue is the result of a changing mRNA-protein relationship across thousands of genes. Changes to any translational or post-translational regulatory processes will change this quantitative relationship. We assert that changes in this correlation between conditions such as health and disease are a result of regulatory changes within the cell^35,36^ and are not solely an artifact of measurement technology.

### Individual Genes Have a Fluid mRNA/Protein Relationship

We investigated the change in a gene’s mRNA/protein relationship by observing a change in correlation between normal and cancer tissues (Figure 2). Our metric for a change in correlation is simply comparing the Spearman correlation coefficient between cancer and normal tissue, called Δ_corr. A positive Δ_corr means that the correlation coefficient in tumor samples is larger than in normal samples, i.e., a protein’s abundance tracks more closely to mRNA abundance. A negative Δ_corr means that the correlation in tumors is smaller than in normal samples. We emphasize that Δ_corr is not the same as the differential expression. A change in correlation may be coincident with differential expression for some genes, as seen in ARID1A in lung adenocarcinoma (Figure 2, middle row). However, a change in correlation is not synonymous with differential expression. For example, the protein and mRNA abundance of ARID1B does not change between normal and tumor tissue; there is no differential expression. However, there is a substantial change in the mRNA/protein relationship, as denoted by a Δ_corr value of ∼0.5378. In normal lung tissue, the mRNA/protein correlation is ∼0.027, but in LUAD the correlation is ∼0.565. (Figure 2, bottom row). Thus, the Δ_corr highlights a change in cellular regulation, distinct from a potential change in abundance.

**Figure 2:**
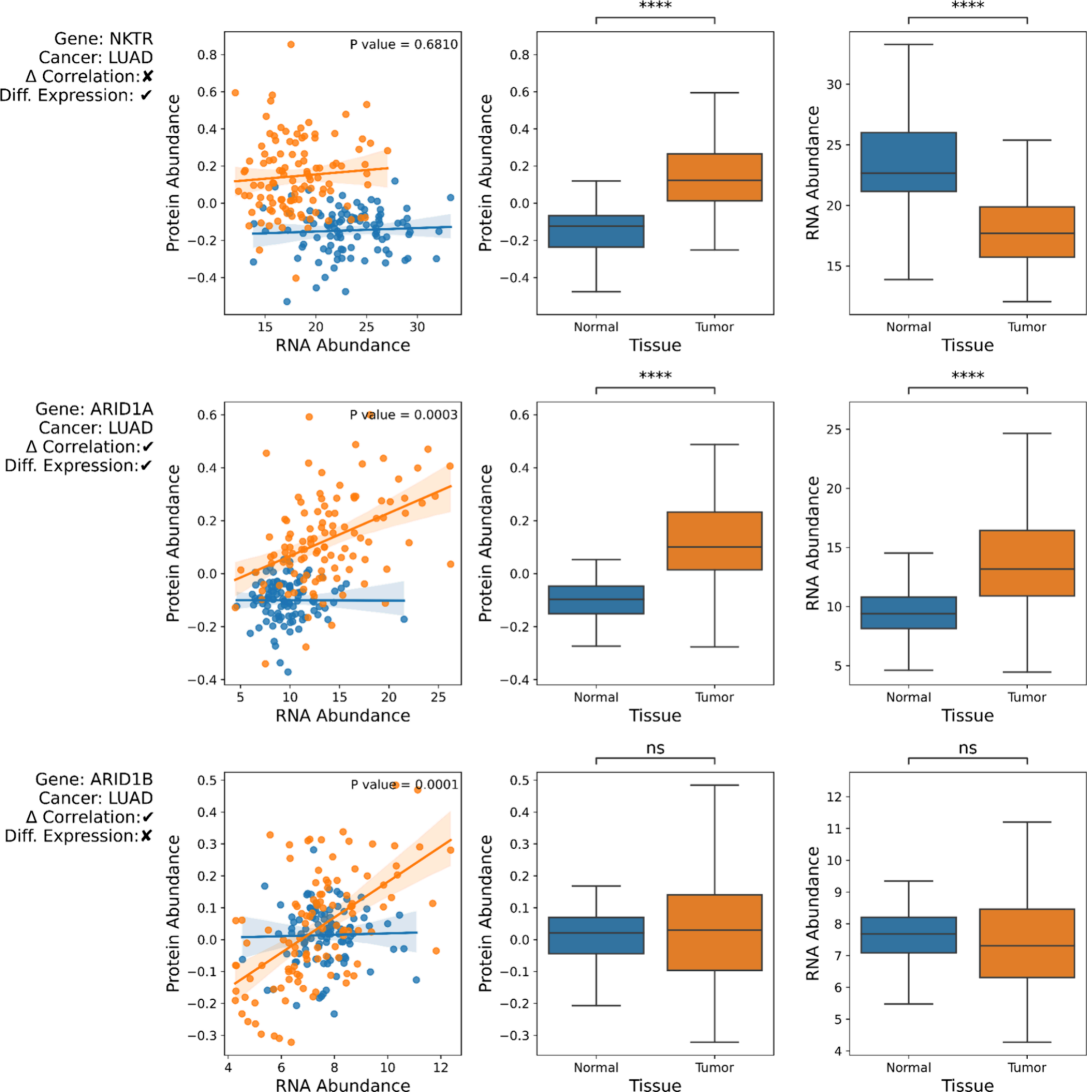
Change in correlation is distinct from change in expression. Three genes from the LUAD data set are shown to demonstrate the independence of Δ_corr and differential expression. The left panel is a scatter plot and regression analysis of each sample’s mRNA and protein abundance (tumor samples in orange, normal tissue in blue). The middle charts are a box plot of the protein abundance with a Wilcoxon rank-sums test for differential expression between tumor and normal tissue. Similar plots on the right are for the RNA abundance. NKTR, top row, has a similar mRNA/protein correlation for both tumor and normal tissue (Δ_corr ∼ 0.056; permutation test BH adjusted *p* ∼ 0.681). However, both the protein and mRNA are differentially expressed (Wilcoxon BH adjusted *p* ∼ 2.707 × 10^–24^). ARID1A, middle row, has a significant change in the correlation (Δ_corr ∼ 0.430; permutation test BH adjusted *p* ∼ 0.003); normal tissue has a noncorrelation (flat blue line), whereas tumor tissue shows a strong positive correlation. Both protein and mRNA are differentially expressed (Wilcoxon BH adjusted *p* ∼ 3.703 × 10^–^^23^). ARID1B, bottom row, has a change in the correlation between tumor and normal (Δ_corr ∼ 0.538; permutation test BH adjusted *p* ∼ 0.001). Normal samples have a noncorrelation (flat blue line), and tumor samples have a positive correlation (positive sloped orange line). This change in correlation is not associated with differential expression (middle and right plots, Wilcoxon BH adjusted *p* > 0.05). The three genes were selected to showcase the diverse cases found in the data.

To understand which Δ_corr values are statistically significant, we calculated a *p*-value using a label permutation test (Figure S3 and Methods). After applying a 5% p-value cutoff (BH corrected) and an absolute threshold of 0.2, we find that a changing mRNA/protein relationship is a prominent feature of cancer. Although the numbers vary depending on cancer type, thousands of genes exhibit statistically significant Δ_corr. Additionally, a majority of genes undergoing this change in correlation do not exhibit changes in abundance (Figure 3).

**Figure 3:**
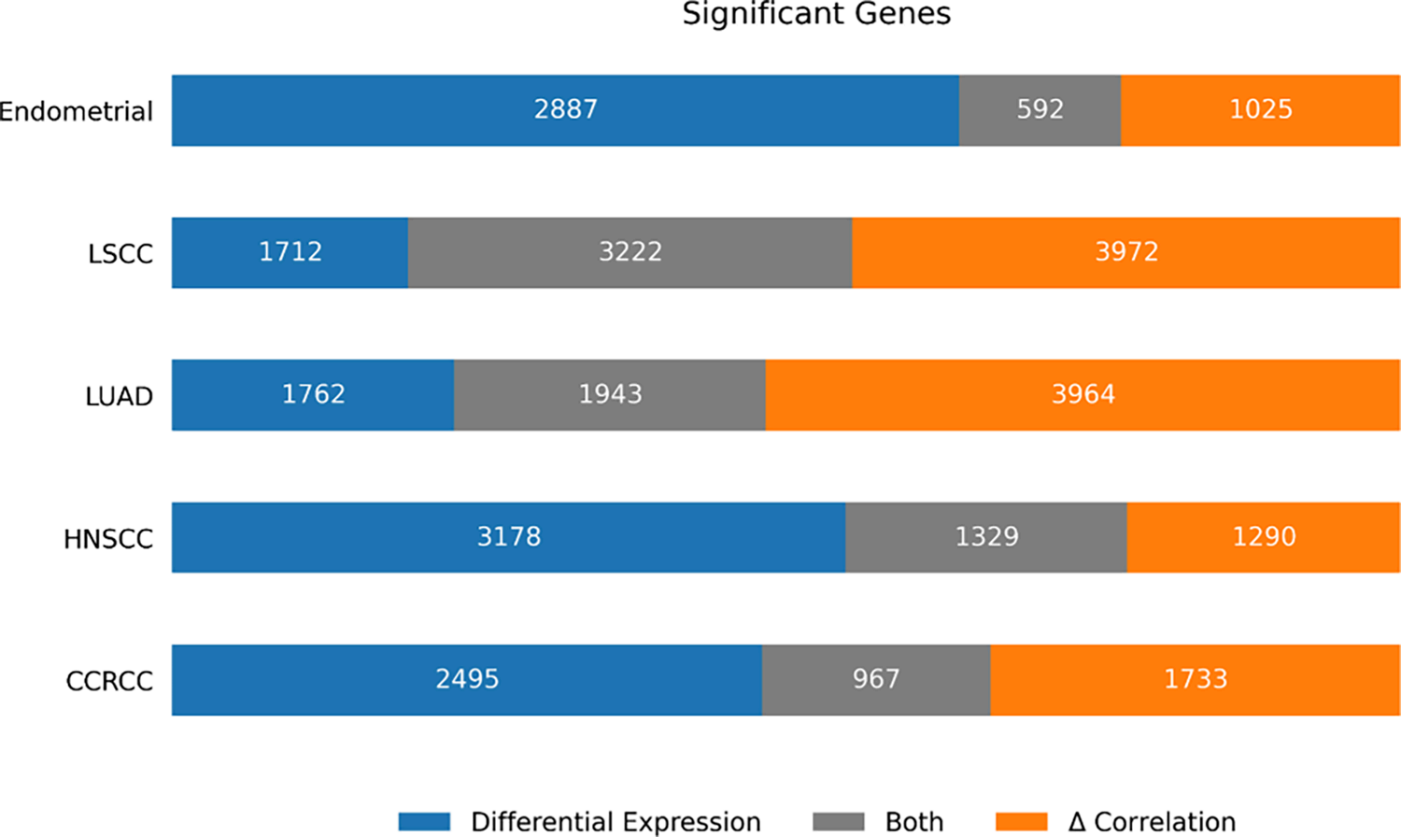
Overlap between differential expression and Δ_correlation. For each gene in each cancer type, we compute both the differential expression and Δ_corr (see Methods). The bar graph shows the overlap between the two metrics. Note that a majority of genes with a statistically significant Δ_corr do not display a statistically significant differential expression.

### Changes in Δ_corr Are Independent of Mutation Status

Given the prominence of somatic mutations in driving the cancer phenotype, we sought to understand the potential role of mutations in the dynamics of the mRNA/protein relationship. We specifically wanted to test whether the observed relationship changes could be attributed to the mutation status of oncogenes and tumor suppressors. For each tumor type, we identified the most frequently mutated genes and compared the mRNA/protein correlation between mutant and wildtype tumors by computing Δ_corr and its associated *p*-value (see Methods). In addition to identifying the effect on the mutated gene, the cis effect, we also calculated these metrics for all trans genes. After multiple hypothesis correction, very few somatic mutations showed an effect, typically fewer than 4 trans genes per mutated oncogene (Figure S2). When compared with the thousands of genes that show a statistically significant change in Δ_corr between tumor and normal tissue, we conclude that the impact of mutation status on the mRNA/protein relationship is negligible.

### Pan-cancer Analysis

Moving beyond single-cancer-type analyses, we next examined this relationship across multiple cancer types to see how a gene behaves in different contexts. For all five cancer types, we calculate the Δ_corr and associated *p*-values for all genes. Genes most commonly exhibit a higher mRNA/protein correlation in tumor tissue, with a positive Δ_corr. However, this is both gene- and tissue-dependent; it is not universal. Reinforcing the idea that the mRNA/protein relationship is fluid and context dependent, genes display a wide variety of patterns across cancer types (Figure 4).

**Figure 4:**
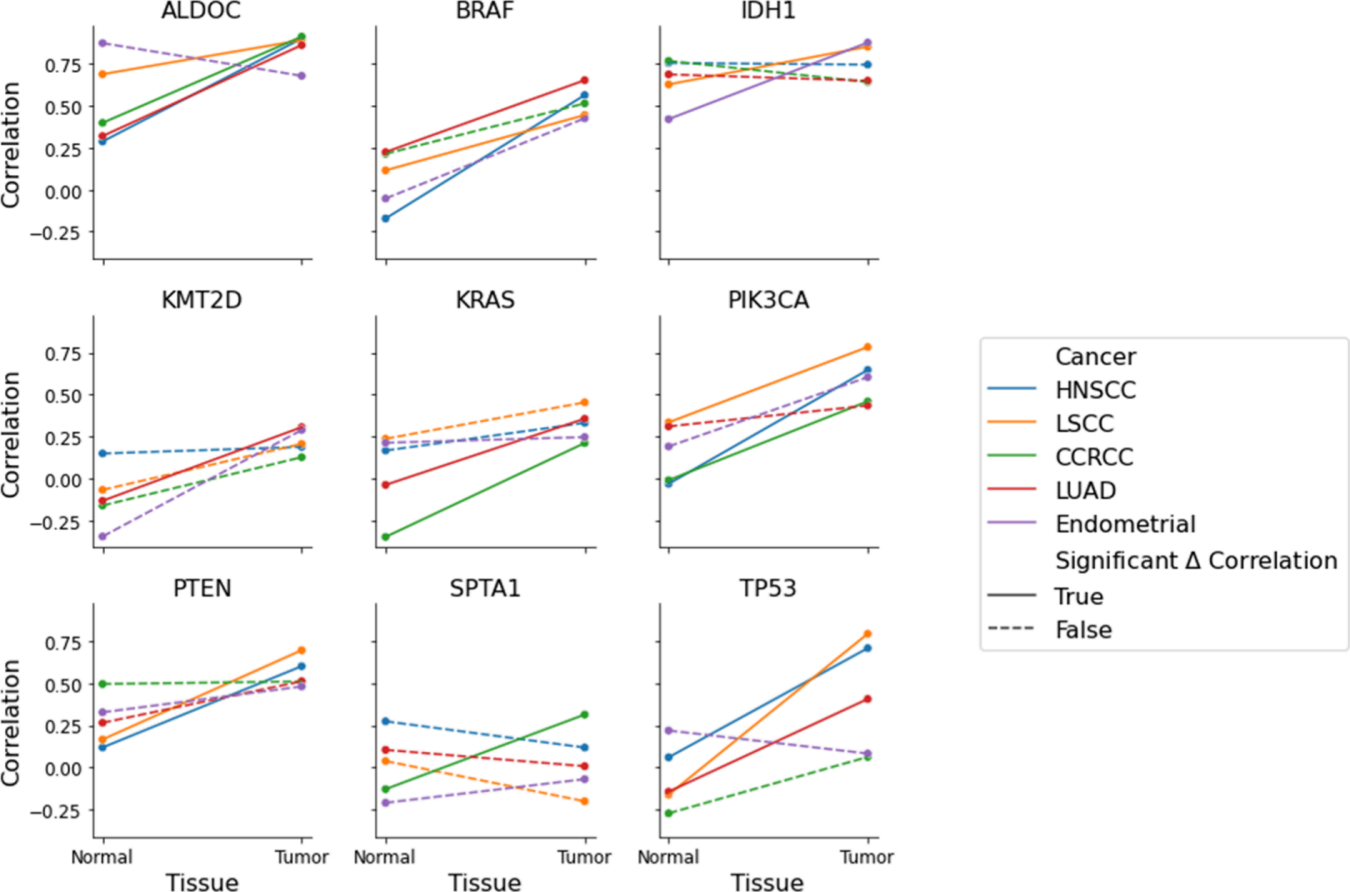
Pan-cancer changes in mRNA/protein relationships. For each gene, we calculate the mRNA/protein correlation in all tissue types (5 tumor and 5 normal tissues). We plot the correlation coefficient and visually connect the tumor/normal for each cohort. Solid lines represent statistically significant Δ_corr, and dashed lines are not statistically significant. Genes often display a variety of changes. The 9 genes in this figure were selected to showcase the different changes in relationship from normal to tumor and how they are different between tissues.

Figure 4 visually represents the diverse patterns of Δ_corr across various genes and cancer types. The BRAF gene has a weak mRNA/protein correlation in all five normal tissues corresponding to a general unresponsiveness of protein abundance to the mRNA levels. For the five cancer tissues, the correlation increases. Additionally, in lung squamous cell carcinoma (LSCC), lung adenocarcinoma (LUAD), and head and neck squamous cell carcinoma (HNSCC), the correlation change is statistically significant. Some genes also exhibit a decrease in the correlation in tumor tissues. SPTA1 has a negative Δ_corr in four of the tissue types we examined; only in ccRCC is the Δ_corr positive. This diversity in regulation is present across all genes and tissues.

### Cancer Hallmarks Are Enriched in Activating Changes in Regulation

After identifying specific genes with a significant change in mRNA/protein correlation via the Δ_corr metric, we examined whether these genes were enriched for cancer-associated biological functions. The analysis of somatic mutation data often identifies a set of genes within pathways related to cell signaling and cell cycle.^4^ Similarly, differential expression analysis of CTPAC data often identifies enrichment for cell cycle, signaling and immune pathways, etc.^19^ As our Δ_corr metric is a new way to describe and identify regulatory changes, we anticipate that these might be concentrated in cancer related genes. Therefore, we performed a gene set enrichment of genes in each cancer to identify pathways and cancer hallmarks that were enriched in changing genes, defined as genes whose Δ_corr had an FDR corrected p-value <0.05 and an absolute threshold of 0.2 for each of the tissues. We observed that the most commonly enriched pathways were metabolic (Figure 5). The most prominent feature of this enrichment analysis is that it identifies metabolic pathways. This is an exciting result as changes in metabolism are well-known phenotype of tumors,^1,37^ yet this hallmark is rarely seen in somatic mutation based analyses.

**Figure 5:**
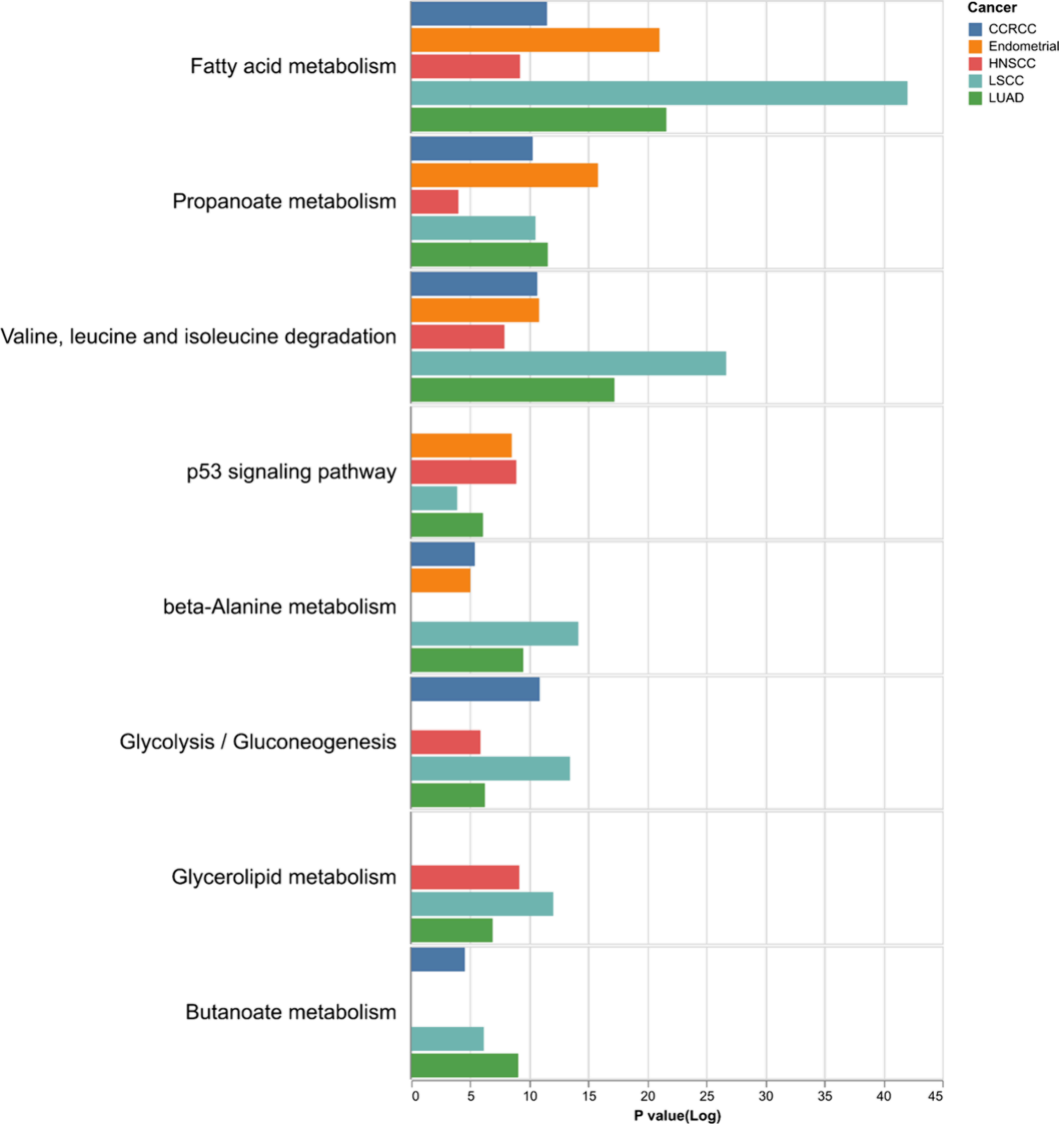
Gene set enrichment analysis of Δ_corr genes. We performed gene set enrichment analysis for genes with a statistically significant Δ_corr value in each tumor type. Plotted are gene sets that were significant across multiple cancers. We note that many metabolic pathways are represented.

## Discussion

In cancer, cells live in an altered regulatory state. Prior research focused on either the presence of mutations or the relative abundance of mRNA or proteins. Here, we introduce a new computational method for integrating transcriptomic and proteomic data. Our metric, Δ_corr, highlights when the quantitative relationship between mRNA and protein changes between conditions, such as a tumor and normal tissue. This metric is distinct from simple differential expression and instead likely points to changes in cellular regulation. We note that the mechanisms that govern these changes are likely to be specific to each gene. Just as gene transcription is controlled by a variety of transcription factors and protein localizations and interactions, we anticipate that the mechanisms controlling the protein-mRNA relationship are multifaceted. As an example of potential mechanisms, we note that the abundance of P53 protein oscillates in the absence of any changes to mRNA abundance, and that these oscillations are coincident with P53 post-translational modification and a negative feedback loop with the ubiquitin ligase MDM2.^38,39^ Moreover, these dynamics are variable by tissue type.^40^ Additionally, in early development of C. elegans embryos and the formation of cell identity, translational repression of mRNA transcripts inherited from the egg is cell type dependent and utilizes mechanisms such as transcript localization, sequestration, and inhibition via RNA binding proteins.^41^

The original hallmarks of cancer^1,2^ categorized cellular dysfunction into several broad biological processes, e.g., cell cycle, angiogenesis, etc. The recent characterizations of tumors via mutation profiles or gene expression often identify genes involved in only some of the hallmarks, most often those related to the cell cycle and cell signaling. The cancer hallmark of altered cellular metabolism has rarely been found. However, the application of our new metric to CPTAC pan-cancer data predominantly identifies genes involved in metabolism.

In addition to creating a new method for integrating proteogenomic data and identifying subtle changes in cellular regulation, understanding the mRNA-protein relationship also has implications on future cancer therapeutics. With the successful creation of mRNA therapies for COVID,^42^ many researchers are beginning to explore mRNA therapies for cancer.^43^ In examining the mRNA/protein relationship in numerous tumor and normal tissues, we note that the utilization of mRNA (i.e., the amount of quantified protein relative to the amount of quantified mRNA) is highly variable across tissues. Thus, the dosing of vaccines may need to be carefully examined across tumor types.

## Supplementary Material



## Notes

Figure S1: single gene correlation of the LUAD cohort; Figure S2: effect of mutation on Δ_corr; Figure S3: permutation based *P* value; and Table S1: pathways from the enrichment tests, showing genes found in multiple cancer types (PDF)
