# Supplementary material for: Activating Cancer Hallmarks through Changes in mRNA/Protein Regulation
Source: J Proteome Res. 2025 Jul 17;24(8):3902–12. doi: 10.1021/acs.jproteome.4c00284 (PMC12322998; doi:10.1021/acs.jproteome.4c00284)
Supplement: Supplementary file 1 [file pr4c00284_si_001.pdf]

Activating cancer hallmarks through changes in mRNA/protein regulation

Jose Humberto Giraldez Chavez[1], Nathaniel Barton[1], Caleb M Lindgren[1], Bryn Mendenhall[1], Benjamin Kimball[1], Samuel H Payne[1]*
1. Biology Department, Brigham Young University, Provo UT 84602 USA
* Correspondence to sam_payne@byu.edu

Supplemental Figures

Figure S1 - Single gene correlation of the LUAD cohort.

Figure S2 - Effect of mutation on Δ_corr.

Figure S3 - Permutation based P-value.

Table S1 - Pathways from the enrichment tests, showing genes found in multiple cancer types.

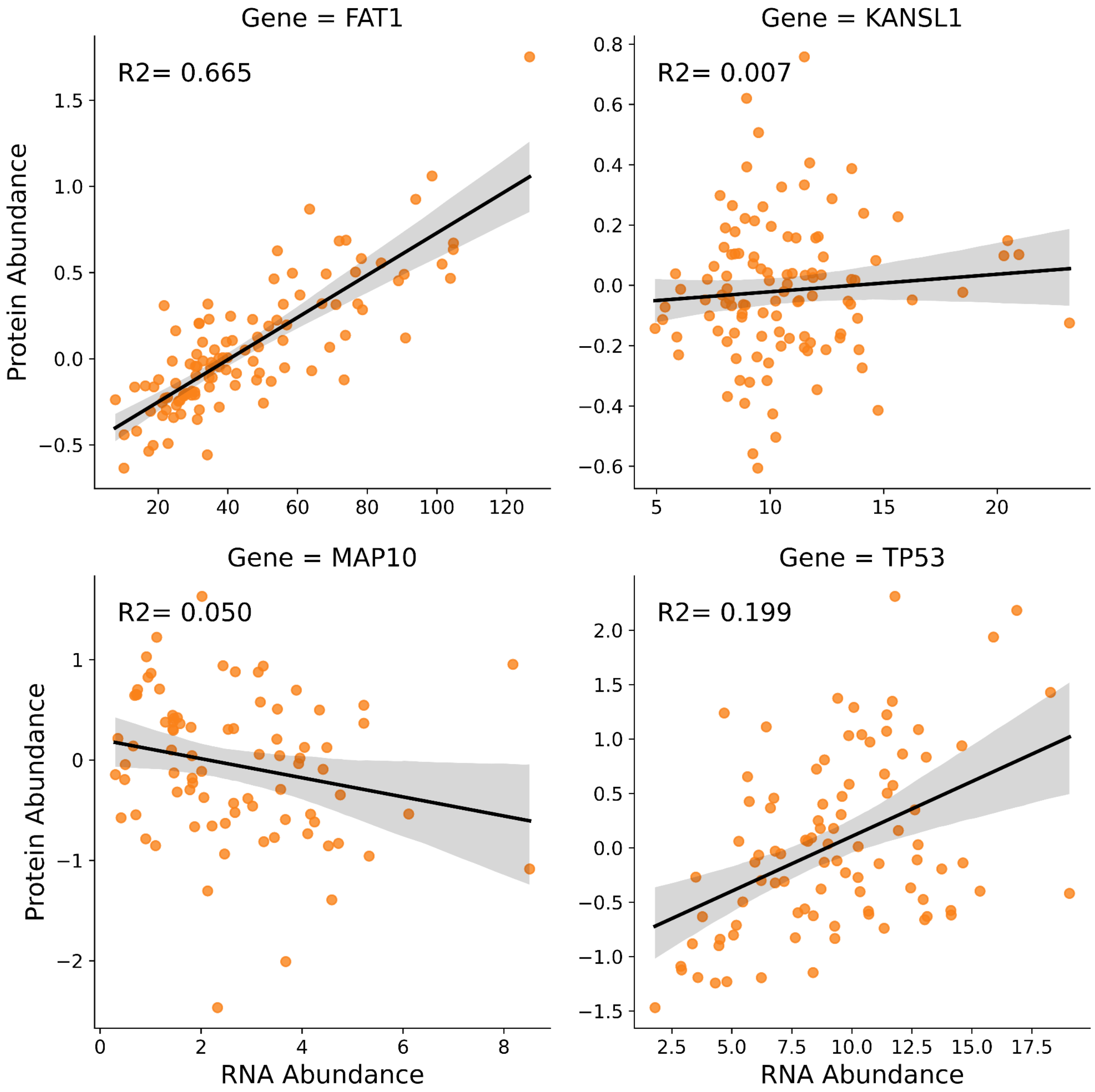

Figure S1 - Single gene correlation of the LUAD cohort. For each gene, the abundance of protein and mRNA abundance is correlated using the Spearman correlation. Each data point represents a single individual within the LUAD cohort.

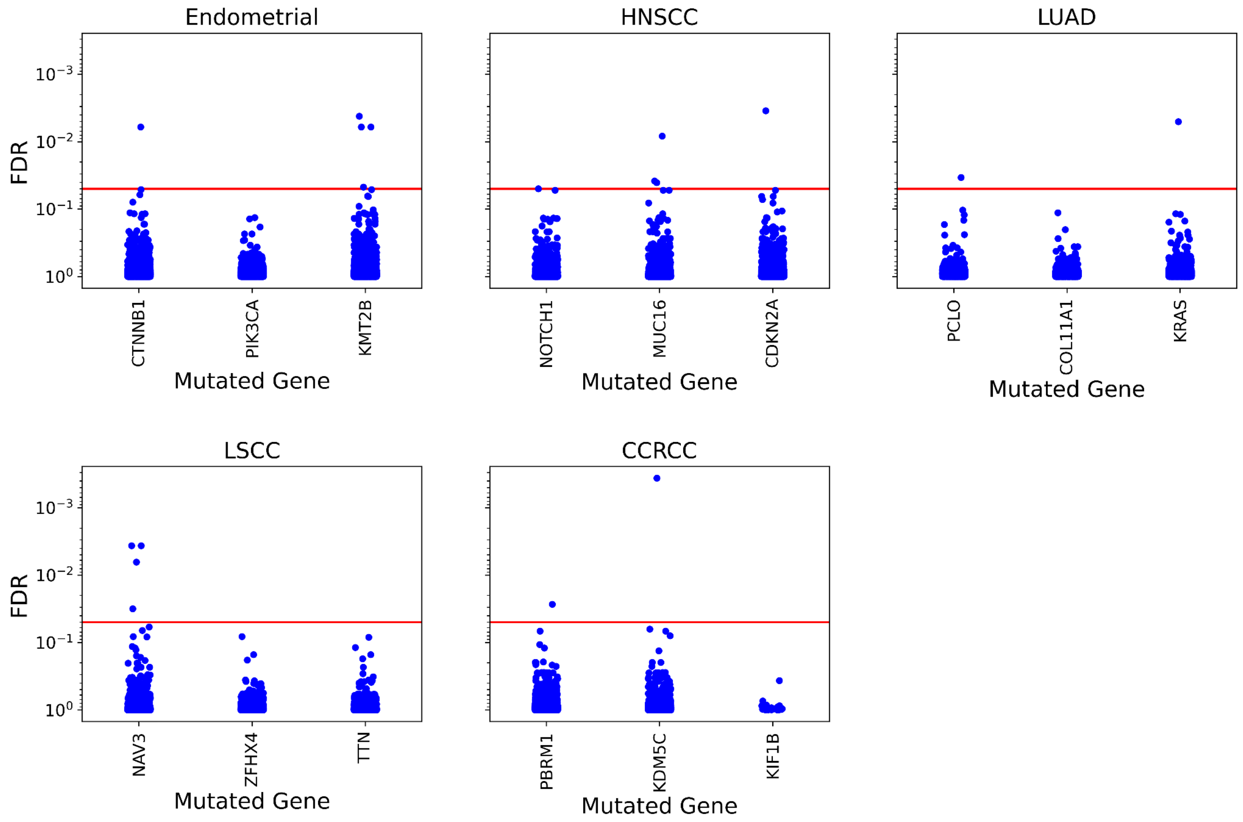

Figure S2 - Effect of mutation on Δ_corr. For each cancer type, we calculated whether a gene mutation affects the protein/mRNA relationship as measured by Δ_corr (see Methods). We show the three most mutated genes, and the BH corrected p-value for all trans genes. A significant p-value (above the red line) indicates that the Δ_corr is partially explained by gene mutation. In general, gene mutation appears to have little to no effect.

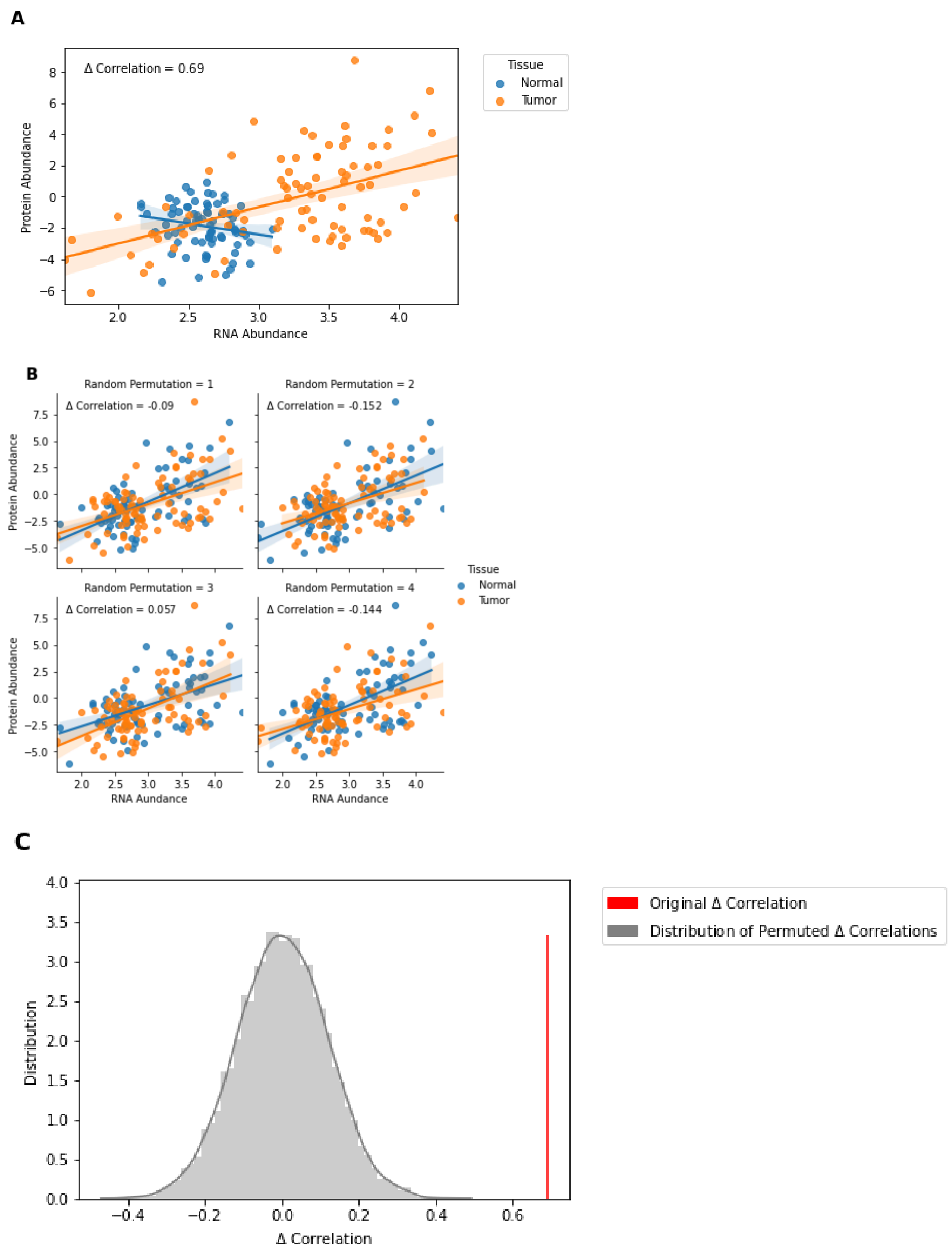
A
Δ Correlation = 0.69
Tissue
Normal
Tumor
Protein Abundance
RNA Abundance
B
Random Permutation = 1
Δ Correlation = -0.09
Random Permutation = 2
Δ Correlation = -0.152
Random Permutation = 3
Δ Correlation = 0.057
Random Permutation = 4
Δ Correlation = -0.144
Tissue
Normal
Tumor
Protein Abundance
RNA Aundance
C
Original Δ Correlation
Distribution of Permuted Δ Correlations
Distribution
Δ Correlation

Figure S3 - Permutation based P-value. We used a permutation label-swap method for determining the p-value of Δ_corr. Panel A shows the true data. Panel B shows four different label randomizations along with their calculated Δ_corr. We performed 10,000 label swaps and aggregated their Δ_corr values into a NULL distribution. We calculate the p-value of the true data by computing the z-score from the NULL distribution (Panel C).

Table S1 - Pathways from the enrichment tests, showing genes found in multiple cancer types.

	Endo	LUAD	LSCC	HNSCC	CCRCC	Total
**AACS**	1	1	0	0	0	2
**ABAT**	0	1	1	0	1	3
**ACAA1**	0	0	1	0	1	2
**ACAA2**	1	1	1	0	0	3
**ACAD8**	1	1	1	1	1	5
**ACADM**	0	1	1	1	0	3
**ACADS**	1	1	1	1	0	4
**ACADSB**	0	0	1	0	1	2
**ACAT1**	0	1	1	0	0	2
**ACAT2**	0	0	1	0	0	1
**ACSF3**	1	0	1	1	1	4
**ALDH1B1**	0	0	1	0	0	1
**ALDH2**	1	1	1	0	1	4
**ALDH3A2**	0	1	1	0	0	2
**ALDH6A1**	0	1	1	0	1	3
**ALDH7A1**	0	1	1	1	0	3
**ALDH9A1**	0	0	0	1	0	1
**BCAT2**	1	0	0	0	0	1
**DBT**	1	0	0	0	0	1
**ECHS1**	1	0	0	0	0	1
**EHHADH**	1	0	0	0	0	1
**HADH**	1	0	0	0	0	1

**HADHB**	1	0	0	0	0	1
**HIBADH**	1	0	0	0	0	1
**HMGCS1**	1	0	0	0	0	1
**IL4I1**	1	0	0	0	0	1
**IVD**	1	0	0	0	0	1
**MCCC1**	1	0	0	0	0	1
**MCCC2**	1	0	0	0	0	1

Valine, leucine and isoleucine pathway

	**Endo**	**LUAD**	**LSCC**	**HNSCC**	**CCRCC**	**Total**
**ACAA1**	0	0	1	0	1	2
**ACAA2**	1	1	1	0	0	3
**ACACA**	1	1	1	0	0	3
**ACADL**	1	1	1	1	1	5
**ACADM**	0	1	1	1	0	3
**ACADS**	1	1	1	1	0	4
**ACADSB**	0	0	1	0	1	2
**ACADVL**	0	1	1	0	0	2
**ACAT1**	0	1	1	0	0	2
**ACAT2**	0	0	1	0	0	1
**ACOX1**	0	0	0	0	1	1
**ACOX3**	1	1	1	0	0	3
**ACSF3**	1	0	1	1	1	4
**ACSL1**	0	0	1	1	0	2
**ACSL3**	0	1	1	0	0	2
**ACSL4**	1	0	1	0	0	2
**ACSL5**	1	1	1	0	1	4

Fatty acid metabolism pathway

	Endo	LUAD	LSCC	HNSCC	CCRCC	Total
**AIFM2**	1	0	0	1	0	2
**APAF1**	0	1	0	0	0	1
**ATM**	0	0	1	0	0	1
**ATR**	0	1	1	1	0	3
**BCL2**	0	1	1	0	0	2
**BCL2L1**	0	1	1	1	0	3
**CASP3**	1	1	1	0	0	3
**CASP8**	0	1	1	0	0	2
**CASP9**	1	1	1	1	0	4
**CCNB1**	1	1	1	1	0	4
**CCNB2**	1	1	1	1	0	4
**CCND1**	0	1	0	1	0	2
**CCND3**	0	0	1	0	0	1
**CCNE1**	1	1	1	0	0	3
**CD82**	1	0	1	0	0	2
**CDK1**	1	1	1	1	0	4
**CDK2**	1	1	1	1	0	4
**CDK6**	0	1	1	1	0	3
**CDKN1A**	0	1	0	1	0	2
**CDKN2A**	1	1	1	1	0	4
**CHEK1**	1	1	1	1	0	4
**CHEK2**	1	1	1	1	0	4

Fatty acid metabolism pathway

	Endo	LUAD	LSCC	HNSCC	CCRCC	Total
**ABAT**	0	1	1	0	1	3
**ACACA**	1	1	1	0	0	3
**ACACB**	0	0	0	1	0	1
**ACADS**	1	1	1	1	0	4
**ACOX1**	0	0	0	0	1	1
**ACOX3**	1	1	1	0	0	3
**ACSS1**	1	1	1	1	1	5
**ACSS2**	1	0	0	0	1	2
**ACSS3**	1	1	1	0	1	4

Propanoate metabolism pathway

	Endo	LUAD	LSCC	HNSCC	CCRCC	Total
**ABAT**	0	1	1	0	1	3
**ACADS**	1	1	1	0	0	3
**ACOX1**	0	0	0	0	1	1
**ACOX3**	1	1	1	0	0	3
**ALDH1B1**	0	0	1	0	0	1
**ALDH2**	1	1	1	0	1	4
**ALDH3A1**	1	0	1	0	0	2
**ALDH3A2**	0	1	1	0	0	2
**ALDH6A1**	0	1	1	0	1	3
**ALDH7A1**	0	1	1	0	0	2
**AOC3**	0	0	1	0	1	2

beta-Alanine metabolism pathway

	Endo	LUAD	LSCC	HNSCC	CCRCC	Total
**ACSS1**	0	1	1	1	1	4
**ACSS2**	0	0	0	0	1	1
**ADH1B**	0	1	0	0	0	1
**ADH1C**	0	1	1	0	0	2
**ADH4**	0	0	0	1	1	2
**ADH5**	0	0	0	1	0	1
**ADH6**	0	0	0	0	1	1
**ADH7**	0	0	1	0	0	1
**ADPGK**	0	0	1	0	1	2
**AKR1A1**	0	0	1	0	0	1
**ALDH1B1**	0	0	1	0	0	1
**ALDH2**	0	1	1	0	1	3
**ALDH3A1**	0	0	1	0	0	1
**ALDH3A2**	0	1	1	0	0	2
**ALDH7A1**	0	1	1	1	0	3
**ALDH9A1**	0	0	0	1	0	1
**ALDOA**	0	1	1	0	0	2
**ALDOB**	0	0	0	0	1	1
**ALDOC**	0	1	1	1	1	4

Glycolysis / Gluconeogenesis pathway

	Endo	LUAD	LSCC	HNSCC	CCRCC	Total
**AACS**	0	1	0	0	0	1
**ABAT**	0	1	1	0	1	3
**ACADS**	0	1	1	0	0	2
**ACAT1**	0	1	1	0	0	2
**ACAT2**	0	0	1	0	0	1
**ACSM2A**	0	0	0	0	1	1
**ACSM3**	0	1	0	0	1	2
**ALDH5A1**	0	0	0	0	1	1

Butanoate metabolism pathway

	Endo	LUAD	LSCC	HNSCC	CCRCC	Total
**ABHD16A**	0	1	0	1	0	2
**AGK**	0	1	1	1	0	3
**AGPAT1**	0	0	1	0	0	1
**AGPAT2**	0	0	0	1	0	1
**AGPAT3**	0	0	1	0	0	1
**AGPAT4**	0	1	1	1	0	3
**AGPAT5**	0	1	1	0	0	2
**AKR1A1**	0	0	1	0	0	1
**AKR1B10**	0	1	1	1	0	3
**ALDH1B1**	0	0	1	0	0	1
**ALDH2**	0	1	1	0	0	2
**ALDH3A2**	0	1	1	0	0	2
**ALDH7A1**	0	1	1	1	0	3
**ALDH9A1**	0	0	0	1	0	1

Glycerolipid metabolism pathway
